# Tetrathiomolybdate alleviates bleomycin-induced pulmonary fibrosis by reducing copper concentration and suppressing EMT

**DOI:** 10.1186/s40001-025-02640-1

**Published:** 2025-05-19

**Authors:** Yajun Wang, Shuyang Chen, Zheng Zhou, Jinjun Jiang, Shujing Chen

**Affiliations:** 1 Department of Pulmonary and Critical Care Medicine, Zhongshan Hospital, Fudan University, Shanghai, China; 2 Shanghai Fifth People’s Hospital, Fudan University, Shanghai, China; 3 Shanghai Respiratory Research Institute, Zhongshan Hospital, Fudan University, Shanghai, China

**Keywords:** Pulmonary fibrosis, Cuproptosis, Tetrathiomolybdate, Bleomycin

## Abstract

Pulmonary fibrosis (PF) is a disease characterized by dysregulated extracellular matrix deposition and aberrant fibroblast activation. Emerging evidence implicates that dysregulated copper metabolism contributed to fibrotic pathogenesis, yet its role and the therapeutic potential of copper modulation remain underexplored. This study investigated the involvement of cuproptosis, a programmed cell death induced by intracellular copper overload, in PF and evaluated the therapeutic efficacy of the copper chelator tetrathiomolybdate (TTM). In a bleomycin (BLM)-induced murine PF model, intratracheal BLM administration elevated lung copper levels, upregulated oligomerized DLAT, and exacerbated fibrosis, as evidenced by collagen deposition, α-smooth muscle actin, and transforming growth factor-beta expression. TTM treatment significantly attenuated fibrotic progression, reduced oxidative stress, and suppressed Olig-DLAT accumulation. In vitro, copper ionophores induced cuproptosis in bronchial epithelial cells, characterized by reduced viability, elevated intracellular Cu⁺, and Olig-DLAT aggregation, which were reversed by TTM. Furthermore, TTM mitigated TGF-β-driven epithelial–mesenchymal transition (EMT) and fibroblast-to-myofibroblast transition (FMT), downregulating collagen-1 and restoring E-cadherin expression. These findings establish cuproptosis as a novel mechanistic contributor to PF and highlight TTM’s dual role in restoring copper homeostasis and inhibiting fibrogenic pathways, offering a promising therapeutic strategy for fibrotic lung diseases.

## Introduction

Idiopathic pulmonary fibrosis (IPF) is a chronic and progressive lung disease characterized by excessive accumulation of extracellular matrix proteins, resulting in tissue scarring, structural distortion, and a subsequent decline in lung function [1]. Among the various models used to study PF, the bleomycin (BLM)-induced fibrosis model in mice is widely recognized for its ability to closely mimic the pathophysiological features of human fibrotic lung disease [2]. BLM, a chemotherapeutic agent, induces lung injury through direct cytotoxic effects and the generation of reactive oxygen species (ROS), initiating a cascade of inflammatory and fibrotic responses [3].

The role of transforming growth factor beta (TGF-β) in the development of fibrosis is well-established [4]. TGF-β is a key fibrogenic cytokine that drives the process of epithelial–mesenchymal transition (EMT), during which epithelial cells lose their characteristics and gain mesenchymal properties, contributing to fibroblast proliferation and accumulation of extracellular matrix proteins [5]. Furthermore, the transition of fibroblasts to myofibroblasts, characterized by the expression of α-smooth muscle actin (α-SMA), marks a critical step in the progression of fibrosis, promoting increased matrix deposition and contractility [6].

Recent studies have highlighted the potential involvement of metal ions, particularly copper, in the pathogenesis of fibrotic diseases [7, 8]. Copper is an essential trace element necessary for various biological processes [9], but can be cytotoxic when dysregulated [10–12]. In physiological conditions, intracellular copper homeostasis is maintained through a tightly regulated process involving absorption via metalloreductases and copper transporter 1 (CTR1) in intestinal epithelial cells, followed by systemic transport via albumin-bound Cu^+^ and hepatic storage or biliary excretion mediated by ATP7A. Within cells, Cu^+^ is distributed to subcellular compartments via chaperones (e.g., CCS, SOD1, and COX17) for enzymatic functions, redox regulation, and mitochondrial respiration, while excess copper is effluxed through ATP7A or stored in metallothioneins to prevent toxicity [13]. Elevated copper levels have been considered to be associated with fibrotic processes, although the mechanisms remain to be further elucidated [14, 15]. Recently, intracellular copper overload has been found to be able to trigger a novel form of programmed cell death, namely, cuproptosis. Cuproptosis is characterized by the loss-of-function of ion–sulfur cluster proteins and the toxic accumulation of lipoylated proteins essential for cellular respiration, research has primarily explored the relationship between cuproptosis and oncological diseases, with some researches attempted to induce cuproptosis in tumors with nanomaterials, offering potential therapeutic insights [16, 17]. Our research has identified the differentially expression of cuproptosis-related genes from patients with severe pneumonia [18], leading us to further explore the potential involvement of cuproptosis and dysregulated copper metabolism profile from a mechanistic approach in other respiratory diseases.

Tetrathiomolybdate (TTM) is a copper chelator with therapeutic potential in conditions associated with copper dysregulation [19, 20]. TTM has been confirmed to be effective by diminishing the production of vascular endothelial growth factor [21], inhibiting tumor necrosis factor alpha [22], and reducing lysyl oxidase expressions [23]. Nevertheless, the exact mechanism through which TTM exert therapeutic function is not yet completely understood.

Therefore, we aims to explore and identify the involvement of dysregulated copper metabolism and thus cuproptosis in the pathogenesis of PF, and to investigate the therapeutic effect of TTM in PF.

## Methods

### Ethics declarations

All animal experiments were conducted in accordance with the protocols approved by the Institutional Animal Care and Use Committee (IACUC) of Wuhan Servicebio. (Approval No. 2024002), aligning with the principles of the ARRIVE guidelines.

### Animal model and treatment

Male C57BL/6 mice (6–8 weeks, weighing 20–25 g) were procured from Wuhan Servicebio and acclimatized under standard laboratory conditions (25 °C, 12 h light/dark cycle, 50–60% humidity) in the Laboratory Animal Center of Wuhan Servicebio with ad libitum access to food and water for 1 week prior to experimentation. Mice were randomly assigned to three groups (n = 5 per group): control (vehicle-treated), BLM (pulmonary fibrosis model), and BLM + Tetrathiomolybdate (TTM).

Pulmonary fibrosis was established via intratracheal instillation of BLM (Cat. No. HY-17565, MedChemExpress, China). Briefly, BLM was dissolved in sterile phosphate-buffered saline (PBS, pH 7.4) to a final concentration of 2.5 U/mL (equivalent to 1.25 mg/kg body weight). Mice were anesthetized using 2.5% avertin (0.15 mL/10 g, injected intraperitoneally) and placed in a supine position. A 24-gauge catheter was inserted into the trachea, and 50 µL of BLM solution (2.5 U/kg) or PBS (Control group) was slowly administered. Post-procedure, mice were monitored for respiratory distress.

Tetrathiomolybdate (TTM, Cat. No. HY-128530, MedChemExpress, China) was administered at 0.2 mg/mouse/day (equivalent to 0.03 mg/mL in drinking water). The BLM + TTM group received a daily oral gavage of TTM (3 mg/kg), starting 24 h before BLM instillation and continuing for 21 days. Vehicle control mice received an equivalent volume of water. All treatments were administered at a fixed time (9:00–10:00 AM) to minimize variability.

### Histological analysis

Mice were euthanized via anesthetic overdose to ensure minimal distress. For histological evaluation, the left lung lobe was selected due to its uniform structural representation of alveolar and interstitial regions. The extracted lung samples were preserved using 4% paraformaldehyde for 24 h. After fixation, samples were encased in paraffin, and then sliced into 5 μm thick sections. These sections underwent staining with hematoxylin and eosin (Cat. No. C0105S, Beyotime, China) for general morphology, Masson's trichrome (Cat. No. C0189S, Beyotime, China) for collagen deposition, and immunohistochemically stained for α-SMA (Cat. No. abs130621, Absin, China) and TGF-β (Cat. No. KHC0131, Proteintech, China) to assess fibrosis markers.

### Immunofluorescence

For immunofluorescence staining, sections were deparaffinized, rehydrated, and blocked. Sections were then incubated with primary antibodies against α-SMA (Cat. No. 14395-1-AP, Proteintech, China) and TGF-β (Cat. No. 91746-2-RR, Proteintech, China) overnight at 4 °C, followed by incubation with fluorescently labeled secondary antibodies. Nuclei were counterstained with DAPI (Cat. No. P0202, Beyotime, China). Fluorescence images were captured using a Confocal Laser Scanning Microscope (FV3000RS, Olympus, Japan).

### Measurement of copper concentration

The concentration of copper in mice lung tissues and cultured cells was measured using inductively coupled plasma mass spectrometry (ICP–MS) calibrated with copper standards. The data were expressed as copper concentration normalized to sample weight (μg/g tissue) or cell number (ng/10^6^ cells). Blanks and quality control samples were included to verify precision and accuracy during each run.

### ROS detection

Detection of reactive oxygen species (ROS) was conducted using a commercially available kit (Cat. No. S0035, Beyotime, China) according to manufacturer’s protocols. Briefly, treated Beas-2b cells were loaded with DCFH–DA probe at 37 °C for 30 min for incubation. Cells were then analyzed using a flow cytometry (BD, FACS Aria III, United States). Further analysis and quantification were conducted using the FlowJo software (10.8.1, FlowJo, United States).

### Cell culture and treatment

Human lung epithelial cells (Beas-2b) were cultured in DMEM medium supplemented with 10% FBS (Cat. No. 12103 C, Sigma, USA), and 1% penicillin–streptomycin (Cat. No. ST488S, Beyotime, China). Cells were treated with CuCl_2_ (Cat. No. 7447-39-4, Sigma, USA) with or without Elesclomol (Cat.No. S1052, Selleck, USA). For fibrosis simulation, cells were treated with TGF-β (Cat. No. HY-P7118, MCE, China) (10 ng/mL) with or without TTM (10 nM) for 48 h. Z-VAD–FMK (Cat. No. HY-16658B, MCE, China) (30 μM) and Necrostatin-1 (Cat. No. HY-15760, MCE, China) (20 μM) were used to inhibit apoptosis and necrosis, respectively. Cell viability was assessed using a Cell Counting Kit-8 (CCK-8) (Cat. No. 40203ES60, Yeason, China).

### RNA extraction and quantitative real-time PCR (qRT-PCR)

Total RNA was extracted from cells and tissue samples using the RNA Purification Kit (Cat. No. RN001, Yishan Biotechnology, China) following the manufacturer’s protocol. RNA concentration and purity were determined using a spectrophotometer (NanoDrop, Thermo Fisher Scientific, USA), ensuring the A260/A280 ratio between 1.8 and 2.0. Complementary DNA (cDNA) was synthesized from 1000 ng of total RNA using a reverse transcription kit (Cat. No. R233, Vazyme, China). qRT-PCR was performed using the QuantStudio 7 Flex Real-time PCR system (Thermo Fisher, USA) and an SYBR Green-based detection kit (Cat. No. Q711, Vazyme, China) following standard procedure. Relative gene expression levels were calculated using the 2^−ΔΔCt^ method, with GAPDH as the internal control.

Primer sequences used in the study are listed below.

**Table:** 

Target	Forward/reverse	Sequence
Human *Col1A2*	Forward	GAGGGCAACAGCAGGTTCACTTA
	Reverse	TCAGCACCACCGATGTCCA
Human *E-cadherin*	Forward	GACTCGTAACGACGTTGCAC
	Reverse	AGACTAGCAGCTTCGGAACC
Human *GAPDH*	Forward	GGAGCGAGATCCCTCCAAAAT
	Reverse	GGCTGTTGTCATACTTCTCATGG
Human *α-SMA*	Forward	CTGGCATTGCCGACCGAATG
	Reverse	GATCCACATCTGCTGGAAGG
Human *Vimentin*	Forward	GACGCCATCAACACCGAGTT
	Reverse	CTTTGTCGTTGGTTAGCTGGT
Mouse *Gapdh*	Forward	TGGCCTTCCGTGTTCCTAC
	Reverse	GAGTTGCTGTTGAAGTCGCA

### Immunoblot

Protein extraction was performed using a RIPA buffer (Cat. No. P0013B, Beyotime, China) supplemented with protease inhibitors (Cat. No. P1005, Beyotime, China) and phosphatase inhibitors (Cat. No. P1081 and P1082, Beyotime, China). Protein concentrations were quantified using the BCA Protein Assay Kit (Cat. No. E112, Vazyme, China). 20 μg of protein per lane were separated on SDS–PAGE gels and transferred onto 0.22 μm polyvinylidene fluoride membranes. The membranes were blocked with 5% non-fat milk in Tris-buffered saline with 0.1% Tween-20 at room temperature for 1.5 h.

The membranes were incubated overnight at 4 °C with indicated primary antibodies at the recommended dilution. After washing three times with TBS-T, the membranes were incubated with host-specific HRP-conjugated secondary antibodies (Cat. No. SA00001 or SA00002, Proteintech, China) for 1.5 h at room temperature. Immunoreactive bands were visualized using an enhanced chemiluminescence detection kit (Cat. No. E433, Vazyme, China. Band intensities were quantified using ImageJ and normalized to the internal control.

All antibodies used in this study are listed below.

**Table:** 

Target	Manufacturer	Cat. No
α-SMA	Proteintech	14395-1-AP
Col1 A1	Proteintech	67288-1-Ig
DLAT	Proteintech	3654-3-RR
Tubulin	Absin	Abs830032
E-cadherin	Absin	Abs149436
Vimentin	MCE	HY-P80371

### Statistical analysis

Continuous variables were presented as the means ± standard error of the mean (SEM). The normality of data distribution was assessed using the Shapiro–Wilk test, and the homogeneity of variances was evaluated using Levene’s test. For comparisons between two groups, Student’s *t* test was used for data meeting the assumptions of normality and homogeneity of variances. Otherwise, the Mann–Whitney *U* test was applied. For multiple group comparisons, one-way analysis of variance (ANOVA) was conducted, followed by Tukey’s post hoc test for pairwise comparisons when variances were homogeneous or Dunnett’s T3 test when variances were unequal. All analyses were performed using GraphPad Prism Version 8.3.0 (GraphPad Software, San Diego, CA, USA). A *P* value < 0.05 was considered statistically significant.

## Results

### Establishment and characterization of a bleomycin-induced pulmonary fibrosis animal model

**Fig. 1:**
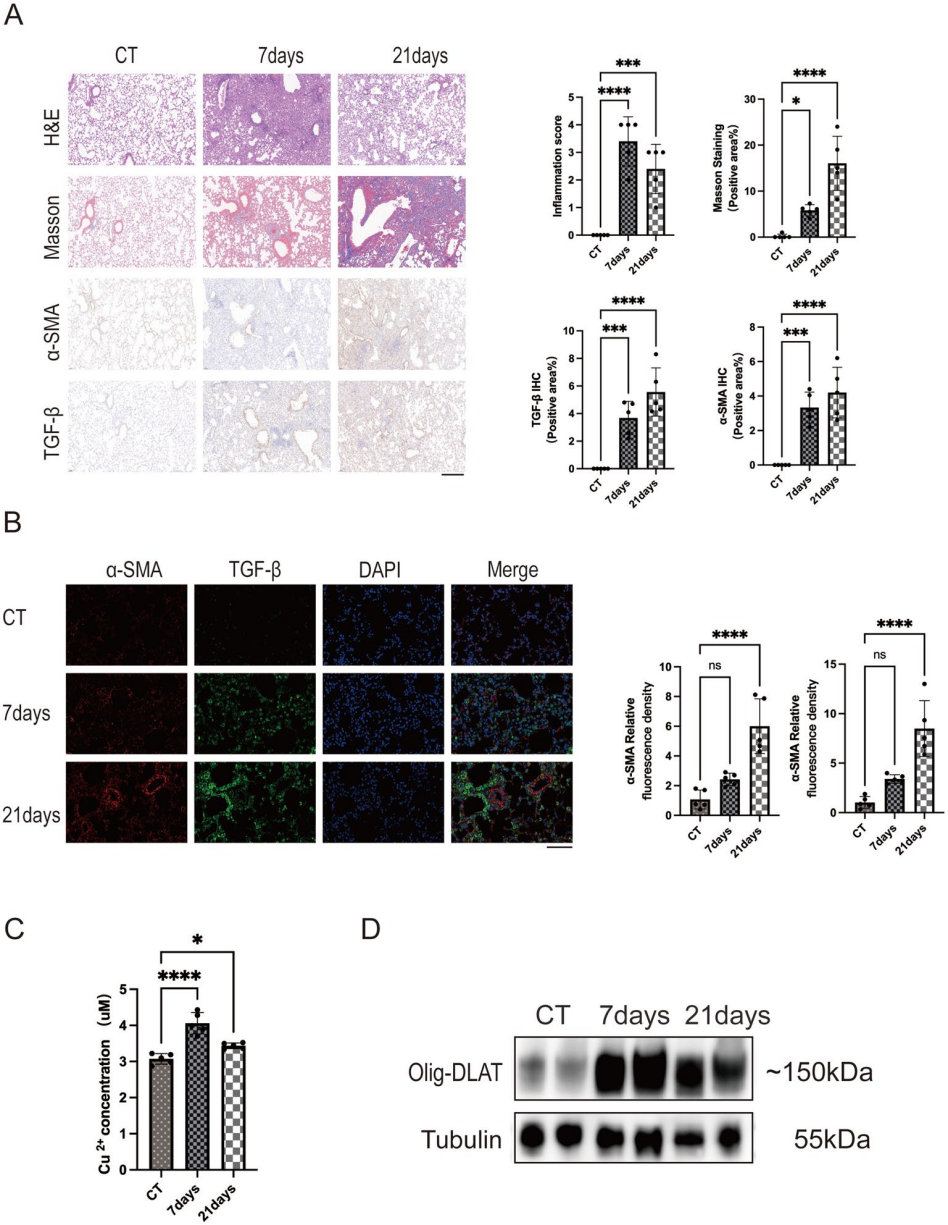
Comprehensive analysis of lung tissue fibrosis, fibrotic markers, copper ion levels, and Olig-DLAT expression in a BLM-induced pulmonary fibrosis mouse model. **A** Representative images of lung tissues stained with H&E, Masson’s trichrome, α-SMA, and TGF-β in control, BLM-treated for 7 days, and BLM-treated for 21-day groups (n = 5), 20×. Scale bar: 250 μm.Quantitative analysis includes Inflammation Score for H&E staining and positive area percentages for Masson’s, α-SMA, and TGF-β staining. **B** Representative immunofluorescence images of lung tissues showing α-SMA (red), TGF-β (green), DAPI (blue), and merged images for control, 63×. Scale bar: 100 μm. Quantitative analysis of relative fluorescence intensities of α-SMA and TGF-β is presented in bar charts. **C** Concentration of Cu^2+^ in lung tissues. **D** Representative western blot images of Olig-DLAT protein expression levels in lung tissues. Data are presented as mean ± SD.*P < 0.05, **P < 0.01, ***P < 0.001, ****P < 0.0001 (*one-way ANOVA*) in this figure

A PF model was established in male 8-week-old C57BL/6 mice through intratracheal injection of BLM, and lung tissue was harvested at 7- or 21-day post-injection for analysis. Left lung sections were fixed and stained with H&E, Masson's trichrome, and immunohistochemical analysis (α-SMA, and TGF-β), respectively (Fig. 1A). At 7-day post-BLM injury, significant pulmonary inflammation was observed, characterized by extensive inflammatory cell infiltration and distortion of normal alveolar architecture. In contrast, by 21-day post-BLM injury, inflammation had subsided, with mild alveolar congestion, exudation, and thickened alveolar walls. The lung injury score decreased compared to the 7-day group. Masson’s trichrome staining, which identifies collagen fibers (blue), revealed minimal collagen formation at 7 days but extensive collagen deposition by 21-day post-injury. Positive Masson’s staining covered approximately 15% of the examined area under light microscopy, confirming successful model establishment. In addition, immunohistochemical staining for α-SMA and TGF-β demonstrated increased expression in both the 7-day and 21-day injury groups, with a more pronounced elevation at 21 days, as indicated by brown–yellow staining positive for α-SMA and TGF-β.

Immunofluorescence assays were further conducted on lung tissue sections, corroborating the previous immunohistochemical findings of elevated expression of α-SMA and TGF-β in pulmonary fibrotic tissue (Fig. 1B). Strong TGF-β positivity was observed in regions adjacent to α-SMA-positive areas, suggestive of a potential correlation. These findings highlight the significant role of TGF-β in the progression of pulmonary fibrosis.

Lung tissue lysates were prepared for measurement of the Cu^2+^ content. Interestingly, BLM-induced lung injury also caused elevated copper ion concentrations in the lung lysates (Fig. 1C). Elevated copper concentration can trigger a novel form of cell death called cuproptosis, leading us to discover whether cuproptosis was involved in the pathogenesis of PF. During cuproptosis, excessive intracellular Cu⁺ binds to lipoylated dihydrolipoamide S-acetyltransferase (DLAT), inducing its oligomerization. Thus, immunoblotting with DLAT as the primary antibody can serve as an indicator of cuproptosis [17]. As expected, oligomerized DLAT (Olig-DLAT), was significantly upregulated following BLM treatment (Fig. 1D). Collectively, these data suggest that BLM treatment disrupted cellular copper metabolism profile, and cuproptosis may be an important mechanism involving the development of fibrosis.

### Copper-induced cellular responses and fibrogenesis

**Fig. 2:**
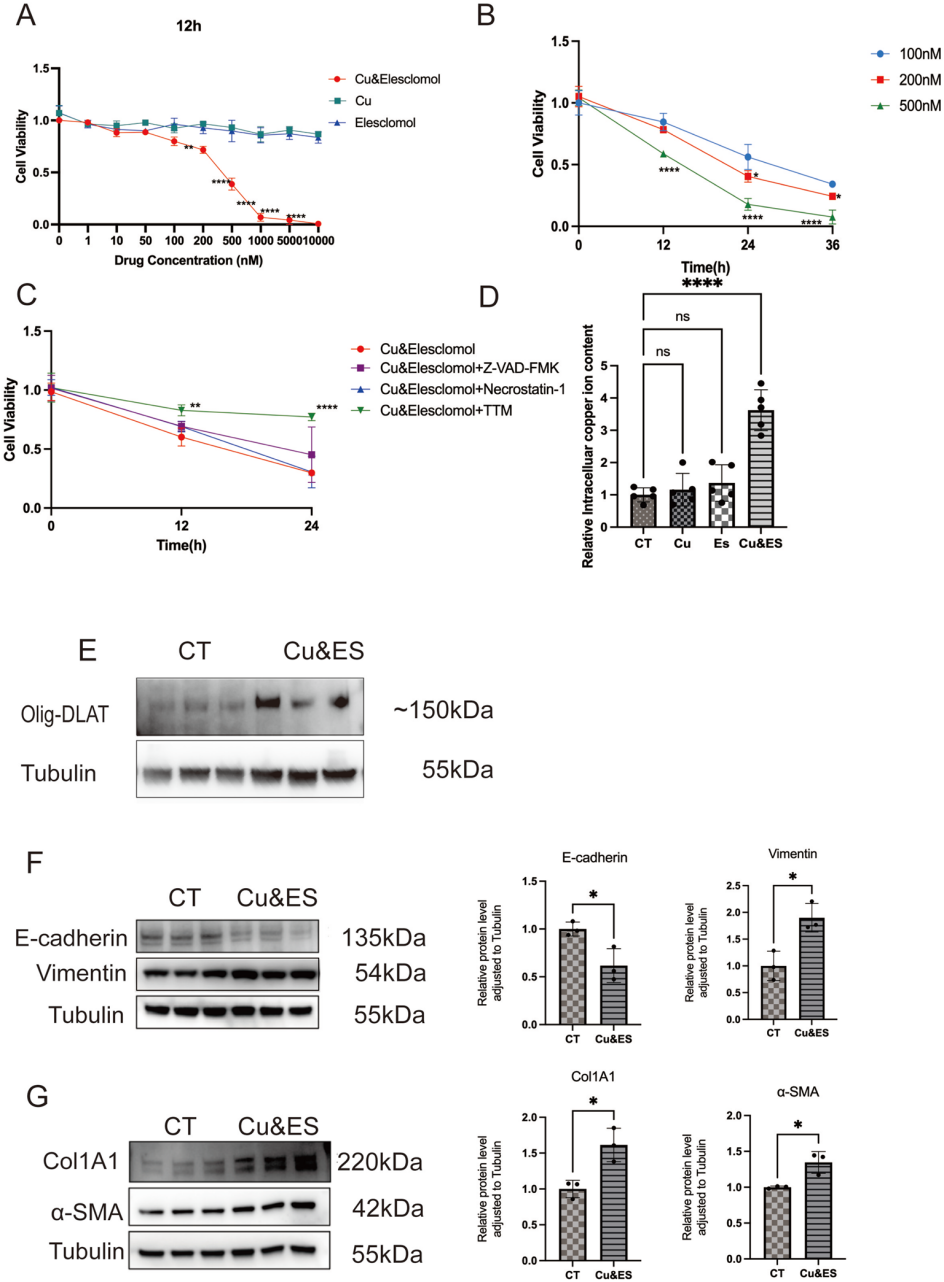
Cytotoxicity, copper ion dynamics, and molecular mechanism analysis of copper ions, elesclomol, and their combination in epithelial and fibroblast cells. **A** CCK-8 assay showing cell viability in three groups: copper ion (Cu) alone, elesclomol alone, and Cu&elesclomol. Cells were treated with increasing concentrations of Cu and/or elesclomol (0, 1, 10, 50, 100, 200, 500, 1000, 5000, 10,000 nM) for 12 h. **B** Time-dependent cytotoxicity of the Cu&elesclomol group at concentrations of 100 nM, 200 nM, and 500 nM after 0, 12, 24, and 36 h, assessed via CCK-8 assay. **C** Effects of inhibitors on Cu&elesclomol-induced cytotoxicity. Treatments include Cu&elesclomol (1:1, 500 nM) alone or in combination with Z-VAD–FMK (30 μM), Necrostatin-1 (20 μM), or TTM (20 μM) for 0, 12, and 24 h, evaluated via CCK-8 assay. **D** Intracellular copper content in cells in four groups: CT, copper ion alone, elesclomol alone, and Cu&elesclomol. **E** Western blot (WB) analysis of Olig-DLAT expression in epithelial cells (Beas-2b) from the control (CT) group and Cu&elesclomol-treated group. **F** WB analysis of epithelial–mesenchymal transition (EMT) markers E-cadherin and Vimentin in epithelial cells (Beas-2b) from the CT group and Cu&elesclomol-treated group, with quantitative analysis. **G** WB analysis of fibrotic markers α-SMA and Col1 A1 in fibroblast cells (MRC-5) from the CT group and Cu&elesclomol-treated group, with quantitative analysis. Data are presented as mean ± SD.*P < 0.05, **P < 0.01, ***P < 0.001, ****P < 0.0001 (*two-way ANOVA or t* tests) in this figure

To ascertain the effects of copper overload on cellular viability, we first attempted to determine whether Beas-2B cells are sensitive to copper ion carrier-induced copper overload. A copper ion carrier, Elesclomol (ES) was added to the culture medium. Initial experiments evaluated the effects of varying copper ion concentrations and their carrier on cellular viability over 12 h (Fig. 2A). The results showed that increasing extracellular Cu^2^⁺ concentrations alone did not affect cell viability. However, the addition of Elesclomol together with CuCl_2_ caused a significant reduction in cell viability, which became progressively pronounced with higher concentrations of the carrier. Next, Beas-2B cells were treated with Cu^2^⁺ and Elesclomol (Cu&ES) at concentrations of 100 nM, 200 nM, and 500 nM for 12, 24, and 36 h (Fig. 2B). These findings suggested that Cu&ES suppressed cell viability in a time-dependent manner.

To verify that the decline of cell viability was attributed mainly to cuproptosis rather than other known cell death pathways, Necrostatin-1 and Z-VAD–FMK were used to inhibit apoptosis and necrosis, respectively. The results indicated that only the copper chelator TTM can reverse the decline in cell viability induced by Cu&ES (Fig. 2C).

Based on the cell viability assay, 500 nM Cu&ES was selected to stimulate Beas-2B cells, alongside an equivalent concentration of CuCl₂, to monitor changes in intracellular copper ion (Cu⁺) levels (Fig. 2D). The results showed that increases in exogenous Cu^2+^ concentration did not alter intracellular Cu^+^ levels, yet the addition of the copper ion carrier significantly elevated intracellular Cu^+^ concentration. These findings indicate that only copper ions entering the cells can influence cell viability.

After the addition of the Elesclomol, the expression of the cuproptosis marker Olig-DLAT was examined. Compared to the control group, Olig-DLAT expression was significantly upregulated in the Cu&ES group (Fig. 2E). This suggests that intracellular copper overload induces cuproptosis.

In addition, 10 nM of CuCl_2_ and the copper ion carrier were added to the culture medium of epithelial cells and fibroblasts, respectively, for 48 h. The results (Fig. 2F) revealed that copper and the copper ion carrier could promote the upregulation of the epithelial marker α-SMA and the downregulation of the mesenchymal marker E-cadherin in epithelial cells. In addition, copper and the copper ion carrier were found to enhance the expression of Col1 A1 and α-SMA in fibroblasts (Fig. 2G), suggesting their transition to myofibroblasts, thereby indicating that copper and the copper ion carrier can promote fibrosis.

### Tetrathiomolybdate protects against bleomycin-induced pulmonary fibrosis and cuproptosis

**Fig. 3:**
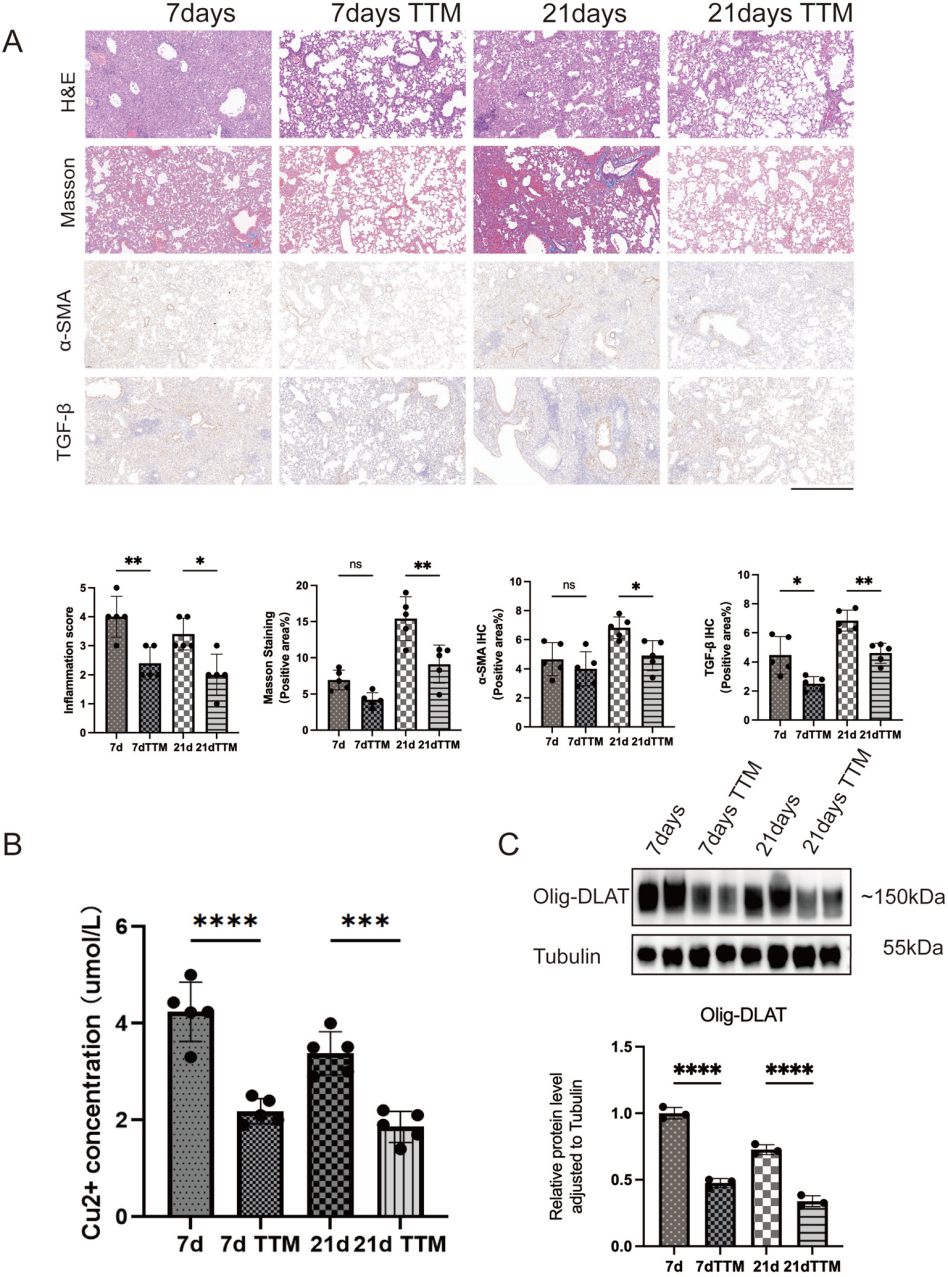
Effects of Tetrathiomolybdate (TTM) on BLM-induced pulmonary fibrosis and related molecular markers. **A** Histological and immunohistochemical analysis of lung tissues from BLM-treated mice (2.5 U/mL, equivalent to 1.25 mg/kg body weight). Groups include BLM treatment for 7 days, BLM with TTM treatment for 7 days, BLM treatment for 21 days, and BLM with TTM treatment for 21 days, 20×. Scale bar: 250 μm. Quantitative analysis includes Inflammation Score for H&E staining and the positive area percentages for Masson’s, α-SMA, and TGF-β staining. **B** Concentration of Cu^2+^ in lung tissues. **C** Representative western blot images and quantitative analysis of Olig-DLAT protein expression levels in lung tissues. Data are presented as mean ± SD.*P < 0.05, **P < 0.01, ***P < 0.001, ****P < 0.0001 (*one-way ANOVA*) in this figure

Following the observation of elevated copper ion concentrations after BLM administration, we began to explore whether the copper chelator tetrathiomolybdate can alleviate BLM-induced lung injury. Histological analysis of left lung sections from treated mice, as illustrated in Fig. 3A, demonstrated that TTM significantly ameliorated BLM-induced lung injury. This included a marked reduction in pulmonary hemorrhage and inflammatory cell infiltration. Quantitative pathological scoring further confirmed that the protective effect of TTM on lung damage was statistically significant.

In addition, Masson’s trichrome staining revealed that TTM markedly reduced collagen fiber deposition in the lungs of the 21-day treatment group, accompanied by a decrease in the expression of α-SMA and TGF-β. In the 7-day injury group, the reduction in collagen fibers and α-SMA expression was less pronounced, likely due to the acute phase of lung injury being less characterized by fibrogenesis. However, TTM still significantly decreased TGF-β levels in this group. Moreover, TTM treatment effectively reduced Cu^2^⁺ concentrations in the lungs of both the 7-day and 21-day groups (Fig. 3B).

Western blot analysis further demonstrated a downregulation of Olig-DLAT in TTM-treated mice (Fig. 3C), providing evidence that TTM mitigates BLM-induced cuproptosis. These findings collectively highlight the therapeutic potential of TTM in alleviating both acute and fibrotic lung injury through copper chelation and modulation of downstream pathways.

### Tetrathiomolybdate mitigates BLM-induced oxidative stress and cuproptosis in lung epithelial cells

**Fig. 4:**
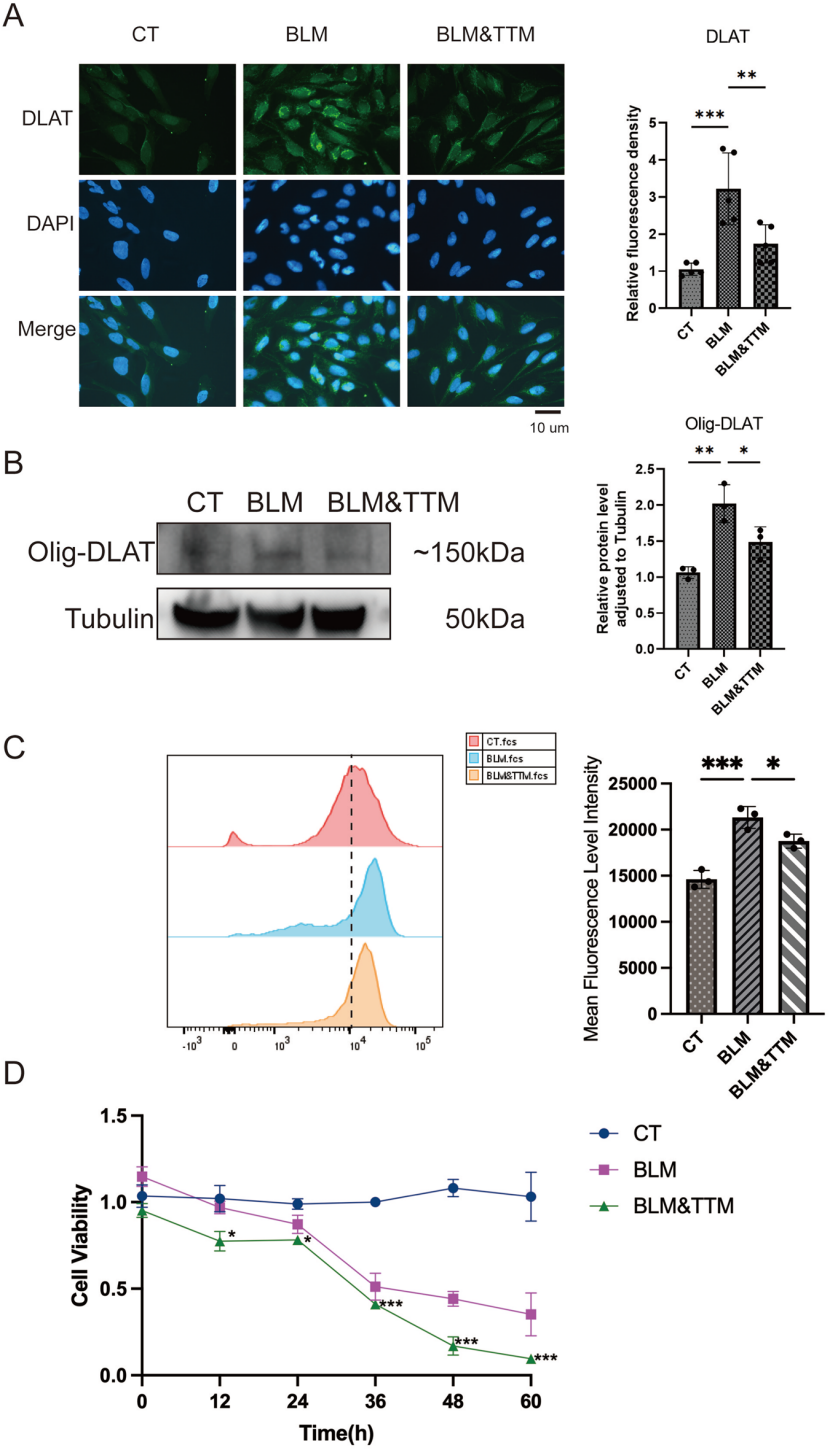
Effects of TTM on DLAT expression, ROS levels, and cell viability in Beas-2b epithelial cells treated with BLM. **A** Immunofluorescence staining of DLAT in Beas-2b cells across three groups: control (CT), BLM-treated for 48 h (BLM), and BLM with TTM (BLM&TTM). DLAT is shown in green, with DAPI staining nuclei in blue and merged images provided. Quantitative analysis of relative fluorescence intensities is presented in bar charts. **B** Western blot analysis of Olig-DLAT expression in Beas-2b cells for the CT, BLM, and BLM&TTM groups. Quantitative analysis of protein band intensities is shown in an accompanying bar graph. **C** Flow cytometric analysis of reactive oxygen species (ROS) levels. Representative histograms are shown, with the mean fluorescence intensity (MFI) of ROS quantified and presented in a bar graph. **D** CCK-8 of Beas-2b cells for the CT, BLM, and BLM&TTM groups at timepoints 0, 12, 24, 36, 48, and 60 h. Data are presented as mean ± SD.*P < 0.05, **P < 0.01, ***P < 0.001, ****P < 0.0001 (*one-way ANOVA or one-way ANOVA*) in this figure

To explore how TTM exerts its protective effects and evaluates its efficacy, Beas-2b cells were used in vitro with BLM added for injury stimulation. Through fluorescence staining, it was observed that BLM stimulation led to an increase in DLAT expression within the epithelial cells, while the addition of TTM reversed this process (Fig. 4A). Furthermore, western blotting experiments revealed that BLM stimulation promoted the expression of Olig-DLAT, which was then ameliorated by TTM (Fig. 4B). These results indicate that BLM can induce cuproptosis in epithelial cells, and TTM possesses protective properties against this process.

In addition, it was found that BLM induces an increase in reactive oxygen species (ROS) (Fig. 4C), while TTM reduces the production of ROS. Concurrently, it was observed that TTM enhances cell viability and can mitigate cell death induced by BLM (Fig. 4D). The findings demonstrate that TTM protects epithelial cells from BLM-induced injury by mitigating cuproptosis, reducing ROS production, and enhancing cell viability.

### TTM reverses TGF-β-induced EMT and FMT in cellular models of fibrosis

**Fig. 5:**
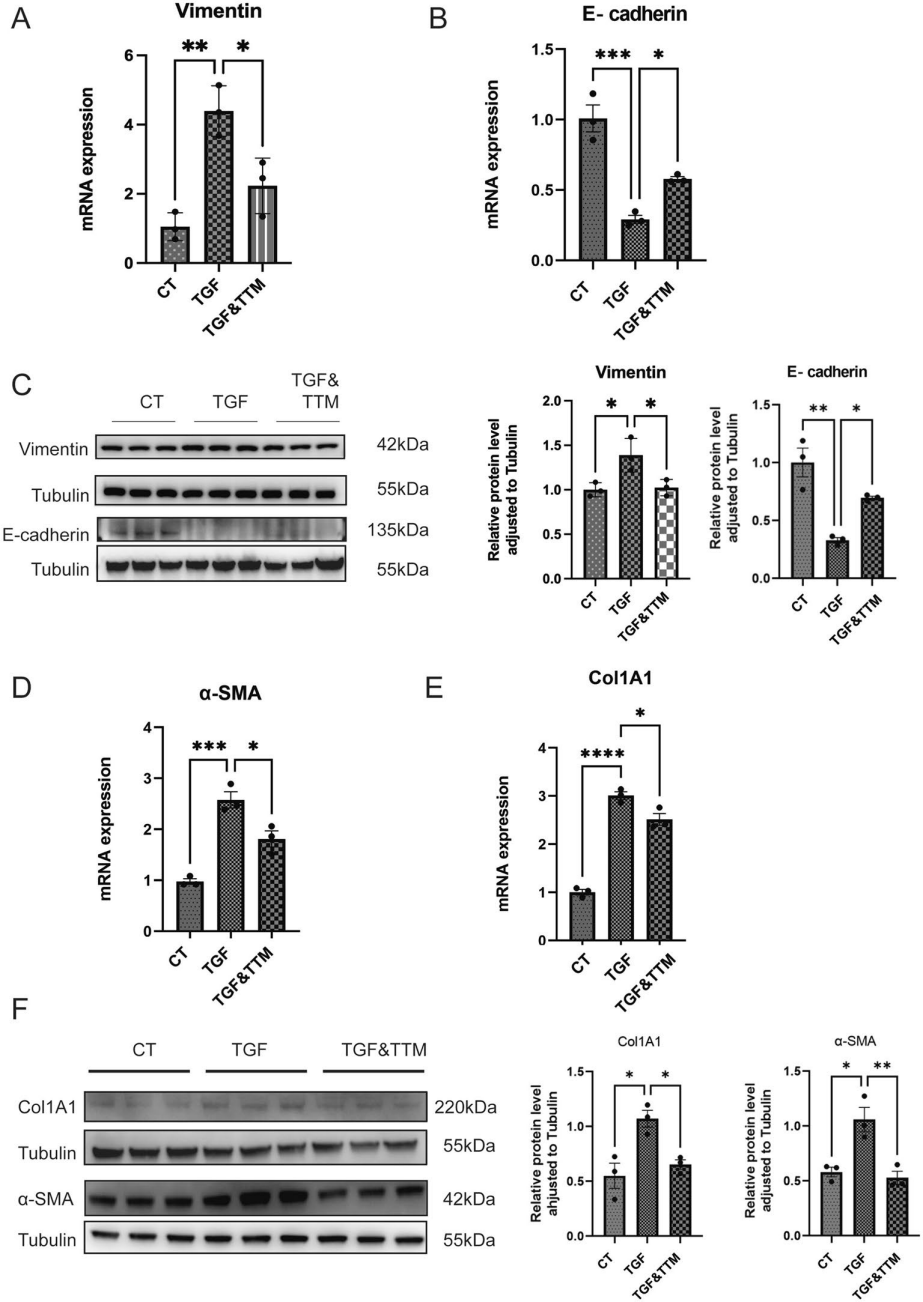
TTM reverses TGF-β-induced epithelial–mesenchymal transition (EMT) and fibroblast-to-myofibroblast transition (FMT). **A**, **B** RT-PCR analysis showing Vimentin and E-cadherin mRNA levels in epithelial cells (Beas-2b cells) across three groups: control (CT), treated with TGF-β (TGF) (10 ng/mL) and TGF-β with TTM (TGF&TTM) for 48 h. **C** Western blot analysis of Vimentin and E-cadherin expression in Beas-2b cells for the CT, TGF and TGF&TTM groups. Quantitative analysis of protein band intensities is shown in an accompanying bar graph. **D**, **E** RT-PCR analysis showing α-SMA and Col1 A1 mRNA levels in in fibroblast cells (MRC-5) across three groups. **F** Western blot analysis of α-SMA and Col1 A1expression in fibroblast cells (MRC-5) for the CT, TGF and TGF&TTM groups. Quantitative analysis of protein band intensities is shown in an accompanying bar graph. Data are presented as mean ± SD.*P < 0.05, **P < 0.01, ***P < 0.001, ****P < 0.0001 (*one-way ANOVA*) in this figure

Epithelial–mesenchymal transition (EMT) plays a critical role in the progression of pulmonary fibrosis (PF). To investigate the effects of copper overload and copper chelation on EMT, we conducted in vitro experiments using TGF-β, a key mediator of EMT, which was found to be upregulated in the fibrosis model during animal studies. TGF-β was added to the culture medium to simulate EMT-related processes and to evaluate the impact of TTM. Treatment with TGF-β led to a significant upregulation of Vimentin and downregulation of E-cadherin at the mRNA level, hallmark changes indicative of EMT. However, these effects were effectively reversed by TTM treatment, suggesting its potential to mitigate EMT (Fig. 5A, B). Western blotting confirmed similar results at the protein level (Fig. 5C), indicating that TTM can reverse the EMT caused by TGF-β.

Fibroblasts were stimulated with 10 ng/mL TGF-β to induce their transformation into myofibroblasts, with TTM added to evaluate its potential inhibitory effects. qRT-PCR analysis revealed that TTM significantly suppressed TGF-β-induced upregulation of α-SMA and Col1 A1 expression (Fig. 5D, E), findings that were further corroborated by Western blot analysis (Fig. 5F). These results demonstrate that TTM effectively reverses fibroblast-to-myofibroblast transition (FMT) induced by TGF-β. Collectively, these findings highlight the potential of TTM as a therapeutic agent in mitigating fibrosis by targeting the FMT process.

## Discussion

The findings of this study highlight the complex interplay between copper homeostasis and the pathogenesis of pulmonary fibrosis, as well as the therapeutic potential of copper modulation in mitigating fibrotic progression.

Copper is a vital trace element essential for the proper functioning of numerous enzymes and proteins within the body [24]. Its importance spans several key physiological processes, including iron metabolism, free radical elimination through its role in the antioxidant enzyme superoxide dismutase, and the maintenance of healthy connective tissue, nerves, and the immune system [25–27]. However, excess copper may lead to cuproptosis, a process characterized by protein aggregation and loss of mitochondrial integrity [17]. In the context of cuproptosis, DLAT plays a central role, which is essential for the mitochondrial tricarboxylic acid (TCA) cycle [17]. The significance of DLAT in cuproptosis arises from its role as a primary target for copper-induced cell death [17].

Copper’s involvement in fibrosis, a process characterized by excessive accumulation of extracellular matrix components, such as collagen in tissues, is complex and multifaceted. The observed increase in pulmonary copper levels in fibrotic lungs may result from dysregulated copper transport and retention. TGF-β signaling and oxidative stress can induce CTR1 upregulation, leading to enhanced copper uptake in fibroblasts and alveolar epithelial cells [28]. In addition, impaired ATP7 A/ATP7B-mediated efflux may contribute to reduced copper clearance [29]. Oxidative stress further enhances metallothionein expression, which sequesters copper within lung tissue [3]. Chronic inflammation and increased vascular permeability could facilitate greater copper influx from circulation. Moreover, lysyl oxidase (LOX), a copper-dependent enzyme required for collagen crosslinking, is upregulated in fibrosis, further contributing to copper accumulation [4, 5]. These mechanisms suggest that targeting copper transport pathways may be a potential therapeutic approach in pulmonary fibrosis.

Copper is a critical cofactor for lysyl oxidase, an enzyme that catalyzes the cross-linking of collagen and elastin fibers in the extracellular matrix [14]. Moreover, copper can catalyze the production of ROS through Fenton-like reactions, leading to oxidative stress [30]. This condition can damage cellular components, including DNA, proteins, and lipids, triggering a fibrotic response as part of the tissue repair mechanism. Oxidative stress also activates fibroblasts, the primary cells responsible for collagen production in fibrotic diseases, further exacerbating the fibrotic process [31]. Recent discoveries have highlighted cuproptosis, a form of cell death driven by copper accumulation [17], as a potential pathway through which copper dysregulation may influence fibrotic disease states. In the study by Tsvetkov et al., Elesclomol, a copper ionophore was used for induction of intracellular copper overload. Similarly, application of elesclomol on the bronchial epithelial cells alone does not induce copper overload or a decrease in cell viability. Only with the presence of copper ions can elesclomol exert its effect on induction of intracellular copper overload, the very premise for cuproptosis. The exact mechanisms by which cuproptosis contributes to fibrosis are still under investigation, but it is thought that the dysregulation of copper homeostasis and subsequent cell death may lead to a cycle of injury and repair, promoting fibrosis [32, 33].In this study, the BLM-induced pulmonary fibrosis model effectively demonstrated the significant impact of copper ions on fibrosis progression, supported by the upregulation of fibrosis markers and the pathological changes observed in lung tissue.

The introduction of TTM as a copper chelating agent revealed its capacity to not only reduce copper levels within the lung tissue but also to reverse key fibrotic processes. A previous study showed that treatment with TTM led to a dose-dependent decrease in serum ceruloplasmin levels, which serves as an indirect indicator of systemic copper levels [34]. In this study, TTM was shown to ameliorate EMT and FMT, critical steps in the development of fibrosis. These effects were corroborated by the downregulation of EMT and FMT markers in response to TTM treatment, indicating a direct link between copper homeostasis and the molecular pathways driving fibrosis.

Furthermore, the study addressed the novel concept of cuproptosis in the context of pulmonary fibrosis. The observed increase in copper levels and the upregulation of the cuproptosis marker, Olig-DLAT, following BLM exposure, suggest that cuproptosis may contribute to the cell death observed in fibrotic lung tissue. The ability of TTM to mitigate these effects further supports the role of copper dysregulation in fibrosis and introduces cuproptosis as a potential target for therapeutic intervention. This study evaluates the preventive potential of TTM in pulmonary fibrosis, considering that copper dysregulation occurs early in the fibrotic process. Pre-treatment strategies have been employed in other fibrosis models, including studies using Nintedanib and Pirfenidone, to investigate whether early intervention can attenuate disease progression. While this study focuses on prophylactic administration, future studies should explore the therapeutic efficacy of TTM when administered after fibrosis onset.

Despite its significant findings, this study has several limitations that warrant further exploration. First, the study primarily utilized a BLM-induced mouse model and bronchial epithelial cell lines, which may not fully capture the complexity of idiopathic pulmonary fibrosis in human patients. Additional studies using primary human cells or patient-derived organoids are necessary to validate the translational relevance of the findings. Second, while the results strongly implicate copper dysregulation and cuproptosis in fibrosis progression, the precise molecular pathways connecting these processes remain incompletely understood. Investigating the interplay between copper-induced oxidative stress, TGF-β signaling, and fibroblast activation could provide deeper mechanistic insights. Third, the long-term safety and efficacy of TTM as a therapeutic intervention remain unclear. Future studies should evaluate the pharmacokinetics, potential off-target effects, and optimal dosing regimens of TTM in preclinical and clinical settings. However, our in vitro experiments used standard EMEM supplemented with 10% FBS, which contains approximately 0.2 µM Cu^2^⁺ as the baseline copper source [35]. This endogenous copper may contribute partially to the observed fibrotic responses; future work should employ defined low-copper media (or serum-free conditions with controlled Cu supplementation) to distinguish effects of baseline versus excess copper.

Addressing these limitations will help advance our understanding of copper homeostasis in fibrosis and support the development of effective therapeutic strategies.

While the current study offers compelling evidence of TTM's efficacy in a BLM-induced model, further research is required to fully understand the mechanisms by which copper influences fibrotic processes and to explore the therapeutic potential of TTM in clinical settings. Ultimately, targeting copper homeostasis may offer a novel and effective strategy for the treatment of idiopathic pulmonary fibrosis and other fibrotic diseases, addressing an unmet need in the management of these challenging conditions.

## Conclusion

This study underscores the significance of copper homeostasis in the pathogenesis of pulmonary fibrosis and introduces a novel therapeutic strategy through the modulation of copper levels. The copper chelator TTM effectively mitigated these effects, reducing oxidative stress, reversing EMT/FMT, and restoring cellular viability. Mechanistically, TTM’s antifibrotic action stems from its capacity to normalize copper homeostasis, thereby interrupting cuproptosis and TGF-β-mediated signaling cascades. These findings open new avenues for research into the role of metals in fibrosis and the potential of targeting metal homeostasis as a therapeutic approach.

## Data Availability

No datasets were generated or analysed during the current study.
